# Changing patterns of cataract services in North-West Nigeria: 2005–2016

**DOI:** 10.1371/journal.pone.0183421

**Published:** 2017-08-17

**Authors:** Nasiru Muhammad, Mohammed Dantani Adamu, Mpyet Caleb, Nuhu Mohammed Maishanu, Aliyu Mohammed Jabo, Muhammad Mansur Rabiu, Covadonga Bascaran, Sunday Isiyaku, Allen Foster

**Affiliations:** 1 Department of Ophthalmology, Usmanu Danfodiyo University Teaching Hospital, Sokoto, Nigeria; 2 Department of Ophthalmology, University of Jos, Jos, Nigeria; 3 Sightsavers, Kaduna, Nigeria; 4 Kilimanjaro Centre for Community Ophthalmology International, Division of Ophthalmology, University of Cape Town, Cape Town, South Africa; 5 Sokoto state eye health programme, Ministry of Health, Sokoto, Nigeria; 6 Helen Keller International, Abuja, Nigeria; 7 Prevention of Blindness Union, Riyadh, Saudi Arabia; 8 Clinical Research Department, International Centre for Eye Health, London School of Hygiene & Tropical Medicine, London, United Kingdom; National Eye Institute, UNITED STATES

## Abstract

**Purpose:**

This study was conducted to assess the impact of the eye care programme on cataract blindness and cataract surgical services in Sokoto, Nigeria over a 12 year period 2005–2016.

**Methods:**

Data from the 2005 population based cross-sectional study of blindness in Sokoto state was re-analysed to obtain baseline estimates of the prevalence of cataract blindness and cataract surgical coverage for persons 50 years and over in Wurno health zone. A population based survey of a representative sample of persons 50 years and over in Wurno health zone was conducted in July 2016. Data on eye health workforce, infrastructure and cataract surgical services between 2005 and 2016 were analysed from relevant documents.

**Results:**

In 2005 the unadjusted prevalence of bilateral cataract blindness (<3/60) in people 50 years and over in Wurno health zone was 5.6% (95% CI: 3.1, 10.1). By 2016 this had fallen to 2.1% (95% CI 1.5%, 2.7%), with the age-sex adjusted prevalence being 1.9% (95% CI 1.3%, 2.5%). The CSC for persons with visual acuity <3/60, <6/60, <6/18 for Wurno health zone was 9.1%, 7.1% and 5.5% respectively in 2005 and this had increased to 67.3%, 62.1% and 34.7% respectively in 2016. The CSR in Sokoto state increased from 272 (1005 operations) in 2006, to 596 (2799 operations) in 2014. In the 2005 survey, couching (a procedure used by traditional practitioners to dislocate the lens into the vitreous cavity) accounted for 87.5% of all cataract interventions, compared to 45.8% in the 2016 survey participants. In 2016 18% of eyes having a cataract operation with IOL implantation had a presenting visual acuity of <6/60 (poor outcome) with the main causes being postoperative complications (53%) and uncorrected refractive error (29%).

**Conclusion:**

Between 2005 and 2016 there was a doubling in cataract surgical rate, a 7 times increase in cataract surgical coverage (<3/60), and a decrease in cataract blindness and the proportion of eyes being couched. However, there remains a high prevalence of un-operated cataract in 2016 indicating a need to further improve access to affordable and good quality cataract surgical services.

## Introduction

The WHO Global Action Plan for Eye Health 2014–2019 aims to achieve increased access to eye care especially in low and middle income countries. It recommends conducting population based surveys to provide an evidence-base for planning and evaluating eye health programmes [1].

Between 2005 and 2014 Sightsavers UK supported the Sokoto state government in Nigeria to develop an eye care programme to improve services in 4 health zones covering all the 23 local government areas (LGAs) of the state. The support covered human resources development, medical equipment and medical supplies.

In 2005 a survey was conducted in the 4 health zones to provide baseline data [2]. To assess the impact of the programme we conducted a follow-up survey in 2016 in one of the four health zones and collated data on the changes in eye care resources and services over the period of the programme.

## Methods

### Study design

The study design has three components. First, the 2005 all-age population based survey [2] in 4 health zones of Sokoto state was re-analysed to obtain data for the 50 years and over age group in Wurno health zone for comparison with the 2016 RAAB survey.

Second, a cross-sectional Rapid Assessment of Avoidable Blindness (RAAB) survey of persons 50 years and over in the Wurno health zone of Sokoto state, Nigeria was undertaken in July 2016.

Third, a review of eye care programme documents [3], including annual reports and evaluation report [4] was undertaken to obtain data on eye health workforce, infrastructure and cataract surgical services over the period 2005 to 2016.

### 2005 survey data re-analysis

The 2005 survey consisted of a sample of persons of all ages from all 4 health zones using stratified cluster sampling [2]. The original data were re-analysed for the population of Wurno health zone, and the prevalence of cataract blindness, cataract surgical coverage, and outcome of cataract surgery in persons 50 years was calculated using Stata 14.

### Rapid Assessment of Avoidable Wurno health zone, 2016

#### Ethical consideration

Ethical committees at the London School of Hygiene and Tropical Medicine and the Sokoto state ministry of health granted approval for the study. Verbal consent was obtained from participants during enumeration; and the rights of individuals to participate or refuse were explained to participating communities.

#### Rapid Assessment of Avoidable Blindness (RAAB) Survey

A minimum sample size of 2706 persons was calculated estimating a blindness prevalence of 10% in persons 50+ years, a precision of 20%, design effect of 1.6 and 95% confidence level.

#### Sample selection

Based upon the last available Census in Sokoto state (2006), an estimation of the 2016 population was made (Table 1). A randomized two-stage cluster sampling strategy was used. In the first stage, 45 clusters were selected from a sampling frame of towns/villages located in the study LGAs using probability proportional to size sampling. In the second stage, compact segment sampling was used to select households in each cluster. A segment was defined as an area comprising 600 persons based on the expected number of persons aged 50 years and over (9.5%) in Sokoto state [5]. All persons aged 50 years and above in each household were enumerated. Survey teams returned for persons absent at the initial visit and to empty houses with eligible subjects; if they were not present for examination they were recorded as absent.

**Table 1:** Target population and sample examined in Wurno health zone, 2016.

Age	Target Population*						Sample Population					
	Males		Females		Total		Males		Females		Total	
	N	%	N	%	N	%	N	%	N	%	N	%
50–59	28,865	26.5	20,227	18.6	49,092	45.1	434	18.0	303	12.6	737	30.6
60–69	17,294	15.9	12,321	11.3	29,615	27.2	522	21.7	454	18.9	976	40.6
70–79	9,657	8.9	6,945	6.4	16,602	15.3	243	10.1	245	10.2	488	20.3
80+	7,554	6.9	5,943	5.5	13,497	12.4	105	4.4	99	4.1	204	8.5
**Total**	**63,370**	58.2	**45,436**	41.8	**108,806**	100.0	**1,304**	54.2	**1,101**	45.8	**2,405**	100.0

* An estimation of the 2016 population was made based upon the last available Census (2006).

#### Survey teams

Two survey teams were trained by a certified RAAB survey trainer. An inter-observer agreement with a minimum kappa score of 0.7 was obtained between the 2 teams in visual acuity measurement, lens assessment and determining causes of blindness and visual impairment at the end of the training period. The first study cluster was jointly conducted with the trainer.

#### Data collection and analysis

After obtaining permission from the head of household the name, age and gender of eligible subjects in each household were recorded into the android-based mRAAB software.

Each eligible subject who was present, after consenting to participate, had a visual acuity measurement and eye examination as described elsewhere [6]. The cause of visual impairment (<6/18) for each eye was assessed based on the coding instructions for WHO/PBL eye examination record [7]. Persons with VA<6/18 caused by cataract were asked why they have not had cataract surgery and up to two barriers were recorded per person. Persons who had received a cataract intervention were asked when and where the intervention took place and if the surgery was free or how much they paid. An ophthalmologist examined each eye to determine the type of cataract intervention and the likely cause of any visual impairment in the operated eyes with VA<6/18.

All findings during the RAAB survey were documented in the android-based data entry software (mRAAB) and checked by the ophthalmologist.

Data were analysed using the RAAB software. Confidence intervals, odd ratios and tests of statistical significance were calculated using Stata 14 software.

### Review of documents

The Sokoto state eye care programme (SECP) project document 2005 [3]; Sokoto state eye care programme annual reports; and an Evaluation Report of the Sokoto state eye care Programme 2014 [4] were reviewed. The Sokoto state eye care project document was developed at a participatory planning meeting held in Sokoto in 2005. The SECP periodic reports are produced by the state ministry of health and shared with all stakeholders; while the evaluation report was conducted by a team of external evaluators. Relevant information on the number and cadre of eye workers, eye care facilities, number of cataract surgeries conducted and sources of funding of the eye care programme over the period 2005 to 2016 were analysed to assess the changes over the 12 years.

### Outcome measures

The following were the main outcome measures:

- Prevalence of cataract blindness: number of people in the sample with <3/60 visual acuity in both eyes due to cataract as a proportion of the sample population;
- Cataract surgical coverage (CSC): the proportion of people who have received cataract surgery as a percentage of all those who could have benefited from cataract surgery (different visual acuity thresholds are provided);
- Cataract Surgical Rate (CSR): the number of cataract operations performed per million population/ year.

## Results

### Re-analysis of the 2005 Sokoto state all age survey for those 50 year and over in Wurno health zone

Of the 4,848 survey participants 648 (13.4%) were aged 50 years and over [2] and of these 190 (29.3%) were from Wurno health zone. The prevalence of all cause blindness in people 50 years and over in all 4 health zones was 11.7% (95% CI: 9.4%,14.4%) and in Wurno health zone 11.6% (95% CI:7.4, 17.0). Cataract was responsible for 48% of all blindness giving a prevalence of cataract blindness of 5.6% (95% CI: 3.1, 10.1).

For Wurno health zone in 2005 the proportion of persons needing cataract surgery that had received a conventional cataract extraction (CSC for persons) for visual acuity categories <3/60, <6/60, <6/18 were 9.1% (3 of 33), 7.1% (3 of 42) and 5.5% (3 of 55) respectively.

### Demographic characteristics of the 2016 survey

In the 2016 survey 89% (2405 of 2700) of the enumerated subjects were examined. Males represented 54.2% of participants.

Compared to an estimate of the population in 2016 based upon the last available Census data in Sokoto state (2006), persons in 50–59 age-group were under-represented in the study sample while the 60–69 age group were over-represented (Table 1).

### Prevalence of cataract blindness

The unadjusted prevalence of bilateral cataract blindness (VA<3/60) with pinhole correction was 2.1% (95% CI: 1.5, 2.7). The prevalence was higher in females, 3.2% than in males, 1.2%–Odds Ratio 2.6 (95% CI: 1.5, 4.9). The unadjusted prevalence of unilateral cataract blindness was 8.9% (95% CI: 7.9%, 10.1%), also higher in females 10.7% (95% CI: 9.0%, 12.7%) than in males 7.4% (95% CI: 6.1%, 9.0%).

The age-sex adjusted prevalence of bilateral cataract blindness was 1.9% (95% CI: 1.3%, 2.5%) and unilateral cataract blindness 8.0% (95% CI: 6.9%, 9.1%), Table 2.

**Table 2:** Age-sex adjusted prevalence of cataract: Wurno health zone 2016.

	<3/60% (95% CI)	<6/60% (95% CI)	<6/18% (95% CI)
**Bilateral cataract**	1.9 (1.3, 2.5)	2.4 (1.8, 3.0)	7.3 (6.2, 8.4)
**Cataract eyes**	5.9 (5.0, 6.8)	7.1 (6.1, 8.1)	12.8 (11.1, 14.4)

### Cataract surgical coverage (CSC)

In 2016 the CSC for persons (<3/60, <6/60, <6/18) in Wurno health zone was 67%, 62% and 35% respectively. For bilateral cataract blindness (VA<3/60) females were significantly less likely to have had cataract surgery compared to males (odds 0.34: 95% CI: 0.16, 0.73. P = 0.002). (Table 3).

**Table 3:** Cataract surgical coverage (CSC persons and eyes): Wurno health zone 2016.

	Persons			Eyes		
**Visual acuity**	**<6/18**	**<6/60**	**<3/60**	**<6/18**	**<6/60**	**<3/60**
**Males**						
**Unoperated cataract (n)**	83	23	16	305	158	129
**Had cataract extraction (n)**	60	60	60	66	66	66
Cataract Surgical Coverage* (%)	**42.0**	**72.3**	**78.9**	**17.8**	**29.5**	**33.8**
**Females**						
**Unoperated cataract (n)**	115	41	35	385	222	188
**Had cataract extraction (n)**	45	45	45	50	50	50
Cataract Surgical Coverage* (%)	**28.1**	**52.3**	**56.3**	**11.5**	**18.4**	**21**
**Total**						
**Unoperated cataract (n)**	198	64	51	690	380	317
**Had cataract extraction (n)**	105	105	105	116	116	116
Cataract Surgical Coverage*(%)	**34.7**	**62.1**	**67.3**	**14.4**	**24.3**	**26.8**

*Cataract Surgical Coverage = Had cataract extraction x 100%

Had cataract extraction + Unoperated cataract

### Visual outcome after cataract interventions

The visual outcome of cataract interventions (couching or conventional cataract extraction) with available and pinhole correction in the 2016 survey are given in Table 4. With available correction, 18% of pseudophakic (cataract extraction with an intraocular lens implantation) eyes had <6/60 (poor) outcome compared to 90% of couched eyes. With pinhole correction 10% of pseudophakic eyes compared to 56% of couched eyes had poor outcome. Table 4.

**Table 4:** Visual outcome after cataract intervention (eyes): Wurno health zone 2016.

Visual outcome	Non-IOL	Pseudophakia	Couched	Total
	N (%)	N (%)	N (%)	N (%)
***Available correction***					
**Good (6/18 or better)**	1 (33.3)	66 (58.4)	0	67 (31.3)
**Borderline (<6/18–6/60)**	0	27 (23.9)	10 (10.2)	37 (17.3)
**Poor (VA < 6/60)**	2 (66.7)	20 (17.7)	88 (89.8)	110 (51.4)
**Total**	**3 (100)**	**113 (100)**	**98 (100)**	**214 (100)**
***Pinhole correction***					
**Good (6/18 or better)**	1 (33.3)	78 (69.0)	13 (13.3)	92 (43.0)
**Borderline (<6/18–6/60)**	0	24 (21.2)	30 (30.6)	54 (25.2)
**Poor (VA < 6/60)**	2 (66.7)	11 (9.7)	55 (56.1)	68 (31.8)
**Total**	**3 (100)**	**113(100)**	**98 (100)**	**214 (100)**

IOL–intraocular lens

Pseudophakia–an eye operated for cataract with IOL insertion.

Of 20 cases of pseudophakic eyes with poor outcome at presentation, surgical and postoperative complications were responsible for 11 cases (53%); and of 27 cases with borderline outcome uncorrected refractive error was responsible for 9 (33%). Table 5.

**Table 5:** Causes of poor and borderline visual outcome in pseudophakic eyes: Wurno health zone 2016.

Cause	Borderline	Poor	Total
	<6/18–6/60	VA<6/60—NLP	<6/18—NLP
	N (%)	N (%)	N (%)
**Co-morbidity**	11 (40.7)	7 (35)	18 (38.3)
**Surgery complication**	4 (14.8)	3 (15)	7 (14.9)
**Refractive error**	9 (33.3)	2 (10)	11 (23.4)
**Long-term complication**	3 (11.1)	8 (40)	11 (23.4)
**Total**	**27 (100)**	**20 (100)**	**47 (100)**

Visual outcome of cataract surgery was poor, with available correction, in 22.7% of pseudophakic eyes operated before 2010, compared to 14.0% in eyes operated after 2012 (P = 0.46).

### Place of surgery

Most patients, 91% (107/118) had their cataract extraction operation in government hospitals and only 4.2% (5/118) were performed in eye camps. Most couching procedures were done in clients’ homes / villages (72%; 71of 98 eyes). Of the 98 couched eyes seen in the 2016 survey 48 (49%) had been couched after 2012.

### Barriers to cataract surgery

Cost (38.3%) was the commonest barrier to uptake of cataract surgery. The second barrier was “denial of treatment” (22.2%) which was due either to denial at an eye care facility (provider-related) or refusal of family members to provide an escort (family-related).

### Trends in cataract surgical rate

**Fig 1:**
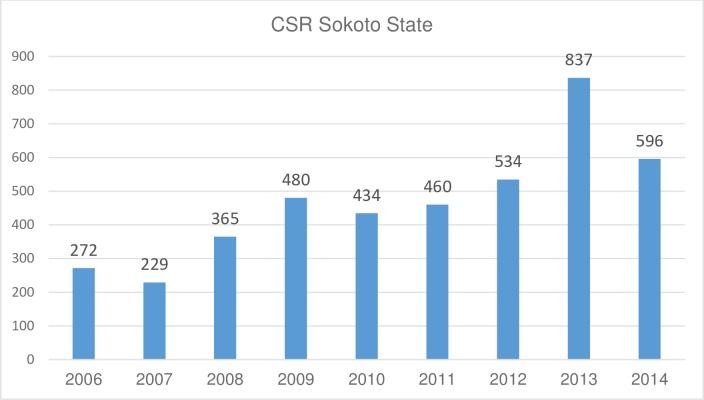
Trends in Cataract Surgical Rate, Sokoto State, 2006–2014.

A total of 17,700 cataract surgeries were performed in Sokoto state between 2006 and 2014 [4]. The annual trend in Cataract Surgical Rate (CSR) for Sokoto state is shown in Fig 1. The CSR was 272/million in 2006 (Cat. Ops. = 1,005) and this had increased to 596/million by 2014 (Cat. Ops. = 2,799) with the highest CSR (837/million) being achieved in 2013.

### Trends in couching

In the 2005 survey, couching was the procedure that had been performed in 87.5% of all previous cataract interventions compared to 45.8% of all previous cataract interventions (98 eyes; 70 persons) seen in the 2016 survey. The presenting visual acuity was poor (<6/60) in 90% of couched eyes, and after providing aphakic spectacles 56% remained with <6/60 acuity.

Using the fee charged for cataract surgery in government hospitals as a measure of comparing the fee charged for couching, 56% of couched eyes in the 2016 survey were provided free or with support from local leaders or well to do persons, 15% were less than hospital fees; and 29% were as much or more than hospital fees.

**Fig 2:**
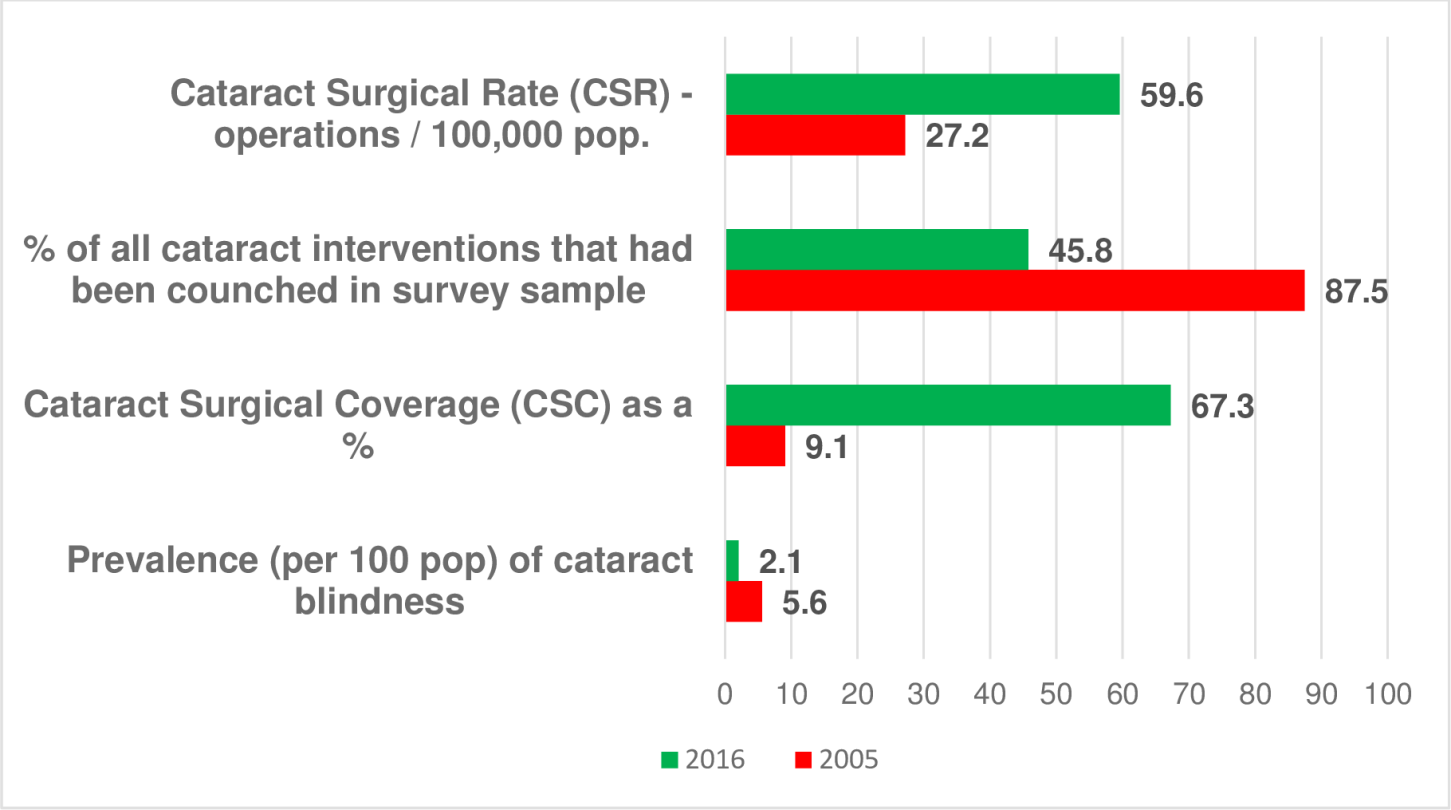
Cataract Service Indicators from the 2005 and 2016 surveys.

The cataract service indicators in 2005 and 2016 are summarised in Fig 2.

### Changes in resources for cataract surgical services

In 2005 there was no ophthalmologist or optometrist in Sokoto state Ministry of Health (MoH) facilities and only 6 ophthalmic nurses; while by 2014 there were 6 ophthalmologists, 36 ophthalmic nurses and 2 optometrists [4]. Programme management staff were deployed by the state MoH and these were reported to be *‘strong and very efficient’*. Programme activities were well coordinated and periodic reports were collated by SECP programme management and shared with planning and research department of the MoH, Sightsavers and programme staff [4].

The number of health facilities providing cataract surgical services increased from 1 in 2005 to 7 in 2014. These eye care services were in general hospitals across the four health zones. The centres provided increased access to eye care for the rural population.

In 2005 the state MoH had an annual budget line for Onchocerciasis and Trachoma vertical control programmes but nothing for general eye services, however by 2016 there was an annual budget line for a comprehensive eye health services programme.

Over the 12 year period the programme has been jointly funded by Sokoto state government, Sightsavers and user fees. Increasingly more patients are paying for their surgeries; in 2009, less than 5% (1 of 22) of cataract surgeries performed were fully paid for by the patient compared to nearly 25% (14 of 57) of those performed after 2013.

## Discussion

### Study design limitations

The 2005 survey was a total population based survey in all 4 health zones of Sokoto state, while for financial and logistical reasons the 2016 survey was only designed for the 50 year and over population and only in one health zone, Wurno. Wurno was chosen as it is the poorest and least accessible of the 4 health zones and therefore improvements seen in Wurno are also likely to have taken place in the other health zones.

The 2005 survey data was reanalysed to provide prevalence data for the 50 year and over population in Wurno zone, however the small sample size means the 2005 estimates have wide confidence intervals.

The high response rate obtained for the 2016 RAAB survey allows generalization of the study findings to Wurno health zone, and gives an insight into the improvements that are likely to be real for the whole state, as shown by the similar baseline data in Wurno and Sokoto state and the increase in state wide CSR. The under-representation of the 50–59 years’ group in the 2016 survey may be due to an economically productive age group being away from their homes, and this under-representation of a younger age group may result in an over-estimate of the overall prevalence of blindness.

### Trends in cataract service delivery: 2005–2016

The increased eye health workforce and infrastructure allowed for an improved provision of cataract surgical services. We found a significant reduction in the prevalence of cataract blindness between 2005 and 2016 which is likely to be attributed to the improved provision of cataract surgical services. The higher prevalence of cataract blindness among females in our study is similar to other surveys [2,8].

The CSC in persons at VA <3/60 increased more than seven-fold from 9.1% % in 2005 to 67.3% in 2016. The CSC at all levels of visual acuity in 2016 was significantly less in females than males which is consistent with findings elsewhere [9, 10, 11]. A study in nearby districts reported women selling more assets or taking loans to access cataract services [12]. In order to improve uptake of services in females, consideration needs to be given to health education and to how surgery can be made affordable for women for example by re-instating a subsidy.

Despite an increase in the overall population of Sokoto state from 3.7 to 4.7 million people, the CSR more than doubled from 272 (1005 operations) in 2006 to 596 (2799 operations) in 2014. While there has been a sustained increase in the CSR in Sokoto state, it still falls short of the recommendation for Africa (2000 operations / million population)[13]. The number of ophthalmologists increased gradually from 1 in 2008; to 3 in 2010 and to 6 in 2012 which undoubtedly contributed to the increase in CSR; however this is still just over 1 ophthalmologist / million population which is a limiting factor to achieving an adequate CSR.

The proportion of persons/eyes with “good” visual outcome following cataract surgery in our study (58% presenting acuity and 69% with pin hole) is higher than the Nigerian national average [14], but below the WHO-recommended standards [15]. There is some evidence for improved outcome in recent years as the proportion of eyes achieving good visual outcome operated within the last 3 years is better than in previous years. Uncorrected refractive error was responsible for 23% (11 0f 47) of operated eyes having an acuity below 6/18 (borderline and poor visual outcome). This is partially due to the lack of biometry equipment and the use of a standard power IOL. Provision of equipment for biometry, and a range of IOL powers are likely to improve visual outcome. Surgical complications and post-operative sequelae were responsible for 38% of operated eyes having <6/18 acuity. Improved monitoring of visual outcome to identify problems with surgical technique and further training in manual Small Incision Cataract Surgery [16] would be appropriate in the current context of available resources in Sokoto state.

At the outset of the programme an annual budget from the MoH and user fees (£5) were introduced to promote financial sustainability. The external donor as well as providing support for equipment and training provided a subsidy for cataract surgery of approximately one-fifth of the cost of cataract surgery. The external subsidy has now ceased so that patients are now requested to pay approximately £25 for cataract surgery. This is a significant barrier in Sokoto state where the poverty rate is 85% [17], and may partially explain the fall in CSR in 2015. The Nigerian National Health Insurance Scheme (NHIS) includes cataract surgery, and this therefore provides an opportunity for the population to join the NHIS and have the costs of cataract surgery covered as is being promoted in Ghana [18].

Our findings indicate an increased acceptance of conventional cataract surgery and a decreased proportion of patients having couching, however this study showed that couching is still commonly practised. It is therefore very important that conventional cataract surgery is made more affordable and available to rural populations, and the community leaders are advised as to the benefits of conventional cataract surgery over couching.

## Conclusion

Our study provides evidence for improvement in cataract services over the last 12 years, as shown by a decrease in cataract blindness with an increase in cataract surgical rate and cataract surgical coverage, combined with improved visual outcome and reduction in proportion of eyes being couched.

However, there are still many people with un-operated cataract and couching is still being practiced. There is a need to further improve the quality of surgical services and make the services more accessible and affordable to the rural poor, especially for women.
